# A Novel Mixed Methods Approach to Understanding Priorities in Emergent Traumatic Brain Injury Anesthesia Care

**DOI:** 10.7759/cureus.79312

**Published:** 2025-02-19

**Authors:** Courtney Gomez, Shuhong Guo, Thitikan Kunapaisal, Christine T Fong, Katie Wolff, Sulayman Jobarteh, Abhijit V Lele, Monica S Vavilala, Marie Angele Theard

**Affiliations:** 1 Anesthesiology and Pain Medicine, Harborview Medical Center/University of Washington, Seattle, USA; 2 Anesthesiology, Prince of Songkla University, Songkhla, THA; 3 Harborview Injury Prevention and Research Center, University of Washington, Seattle, USA

**Keywords:** anesthesia for brain surgery, anesthesiology practice, health priorities, quality assessment in healthcare, traumatic brain injury

## Abstract

Introduction

Traumatic brain injury (TBI) has a high mortality rate. Given the limited evidence regarding optimal anesthesia care for patients with TBI, we elicited anesthesiology provider perspectives on priorities for improving emergent TBI anesthesia care through mixed methods.

Methods

We elicited survey and focus group responses from 177 anesthesiology attendings, nurse anesthetists, and residents. Textual data quantified word characteristics (frequency, repeated words and percentage) by word cloud generation and iterative development of common themes by inductive reasoning. Themes weighted on the frequency of phrases or words were analyzed within another word cloud and classified as structure, process, and outcome measures. A Pareto diagram of themes identified high interest content categories.

Results

In triangulation, the leading 20% of themes were classified into Agency for Healthcare Research and Quality (AHRQ) domains. Twenty-three (13%) survey responses and two focus group data (27 participants) were examined. “Time” was the largest word by word cloud and the most common word (3.57%). Inductive analysis produced 28 content categories (“timeliness” 28.07% most common theme), classified into 11 structure-type, 15 process-type, 2 outcome-type, and no balance quality improvement categories. There were no content categories classified into the balance-type quality measure. A Pareto diagram indicated “timeliness,” “standardization,” “hemodynamics,” and “communication” as important themes. Leading AHRQ domains were “effective, equitable, timely, and safe.”

Conclusion

Word cloud, inductive reasoning, and use of the Pareto diagram identified many opportunities for improving emergent TBI anesthesia care in our institution.

## Introduction

There is a large traumatic brain injury (TBI) burden, annually affecting approximately 60 million people worldwide. In the USA alone, the National Institutes of Health (NIH) spends approximately $64 million annually on TBI research [1]. Yet, TBI-related mortality remains high [2]. Many patients with TBI require and receive anesthesia care for emergency neurosurgery and/or extracranial injuries, but there is limited evidence to guide the emergent anesthetic management of patients with TBI [3-5].

The Brain Trauma Foundation guidelines, which provide evidence-based recommendations for the care of patients with severe TBI, largely refer to the intensive care unit phase of care [5]. Published data from anesthesia care are few, observational, and single center. One study suggests a large secondary insult burden requiring an escalation in postoperative care, but strong evidence is lacking for the anesthesia phase of care [6]. Anesthesiologists are well positioned to help prevent secondary injury during anesthesia care, but information is lacking as to how to achieve this goal, largely due to the lack of anesthesia research in TBI. There is also a paucity of knowledge on anesthesia care priorities, which may inform research question development. Since intraoperative anesthesia care affects perioperative outcomes, knowledge of how to improve anesthesia care of patients with TBI is critical for developing strategies for improving perioperative and hospital-level outcomes. While the time patients with TBI requiring emergent anesthesia care is less than that spent subsequently in the intensive care unit, the physiological perturbations experienced by patients with these critical injuries are potentially life-threatening, making optimizing anesthesia care highly relevant to promoting favorable patient-level outcomes. To address this unmet gap in practice, we sought to understand anesthesiology provider perspectives on priorities for improving delivery of emergent TBI anesthesia care and identifying high-priority content categories suitable for the development of quality indicators and metrics in TBI anesthesia care.

This article was previously presented as a meeting abstract at the Society for Neuroscience in Anesthesiology and Critical Care meeting in September of 2023, and the meeting abstract was published in the Journal of Neurosurgical Anesthesiology in October of 2023 following the presentation (DOI:10.1097/ANA.0000000000000935).

## Materials and methods

Harborview Medical Center is the only level I trauma center in Washington, Wyoming, Alaska, Montana, and Idaho and admits approximately 6,000 injured patients annually, of whom approximately 400 are admitted to the neurocritical care service with TBI and 200 undergo craniotomy/craniectomy [7]. This study was deemed exempt by the Institutional Review Board of the University of Washington (IRB STUDY00020129). The hospital department leadership approved this quality improvement (QI) initiative, and discussions occurred during a scheduled department meeting. We used a survey with free-text response options and formed two focus groups to examine anesthesiology attending physician, resident anesthesiology provider, and nurse anesthetist perspectives between January and April 2023. We iteratively developed an anonymous online REDCap survey among the project team with four free-text response questions, which we administered to 177 hospital’s anesthesiology providers (43 attending physicians, 50 nurse anesthetists, and 84 resident physicians) [8,9].

Quantitative data were examined using Excel (Microsoft Corp., Redmond, WA). Textual data from free-text survey responses and transcribed meeting notes were analyzed quantitatively by counting words and repeat word frequency and the percentage of the sum of words [10]. Based on the Oxford English Corpus (OEC) analysis of two billion English words to determine the 100 most frequently written English words, the most common English words that were deemed unrelated to this work were excluded [11]. Of the remaining words, textual data were then analyzed utilizing a free, online, artificial intelligence word cloud generator, where higher frequency of word count results in a visually larger word to inform subsequent inductive reasoning [12]. Then, survey text data and transcribed notes from focus group discussions underwent inductive content analysis to identify common content themes, developed through manual examination of survey textual data, transcribed notes from group discussions, and examination of the word cloud generated from these data to identify repeated phrases consistent with identifiable themes within the data [13].

**Figure 1:**
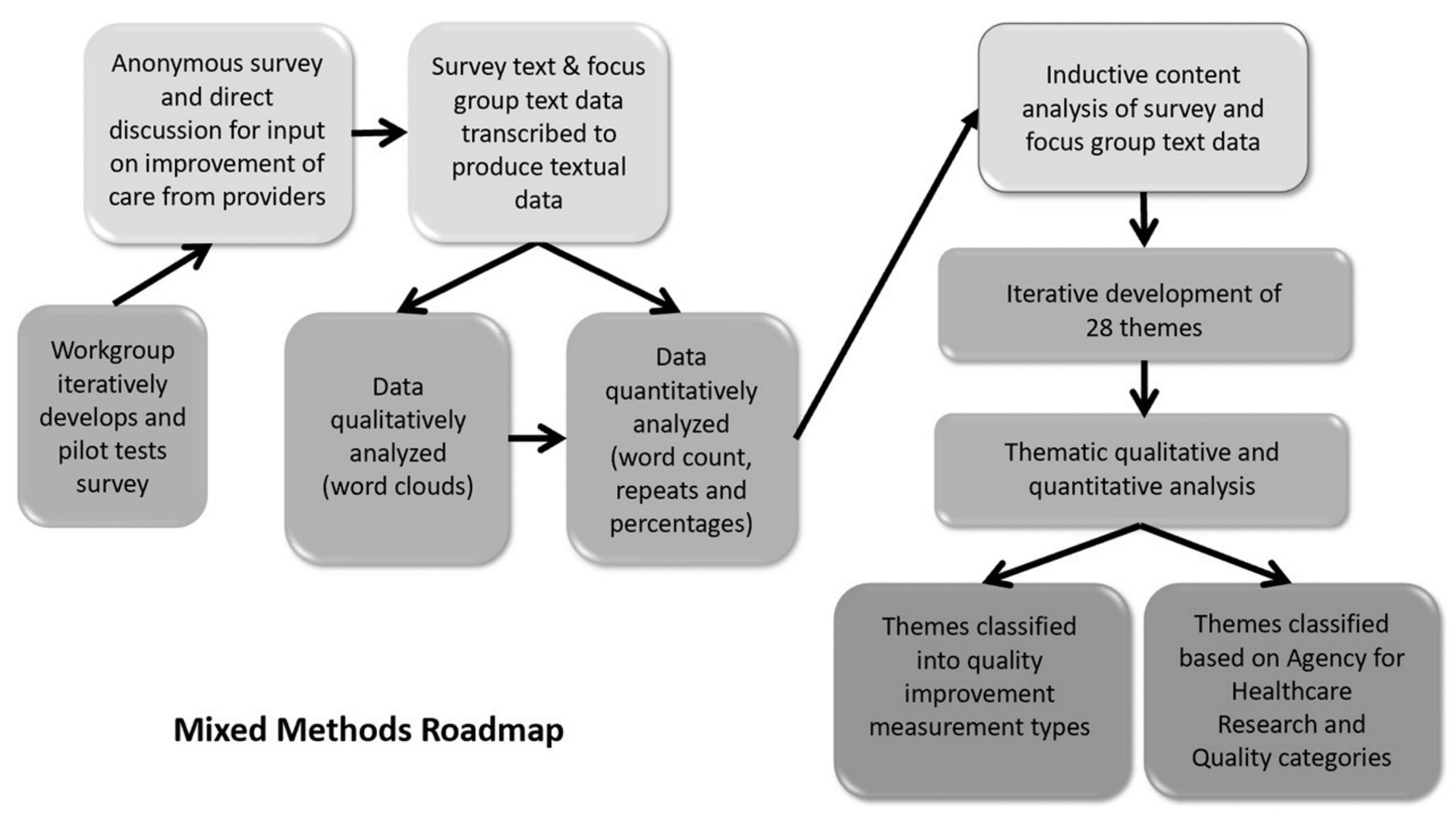
Mixed methods roadmap QI, quality improvement; AHRQ, Agency for Healthcare Research and Quality

Then, in triangulation, themes were weighted on the frequency of phrases or words and graphically analyzed within another word cloud [14]. Resulting themes were classified into structure, process, outcome, and balance measures [15]. A Pareto diagram identified the leading 20% of the themes to improve the quality of care of patients with TBI. Finally, in triangulation, the project team ranked each of the leading 20% of themes into Agency for Healthcare Research and Quality (AHRQ) domains (Figure 1) [16].

See the Appendix for focused stem and survey questions.

## Results

**Figure 2:**
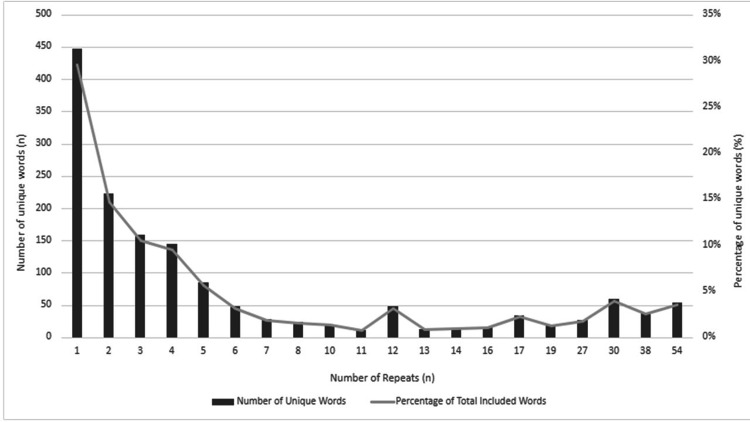
Number of repeats and percentage of unique words Number of repeats (n), number of unique words (n), and percentage (%) of unique words in all textual data from survey and group discussions of traumatic brain injury anesthesia care with anesthesiology providers (attendings, residents, and nurse anesthetists).

**Figure 3:**
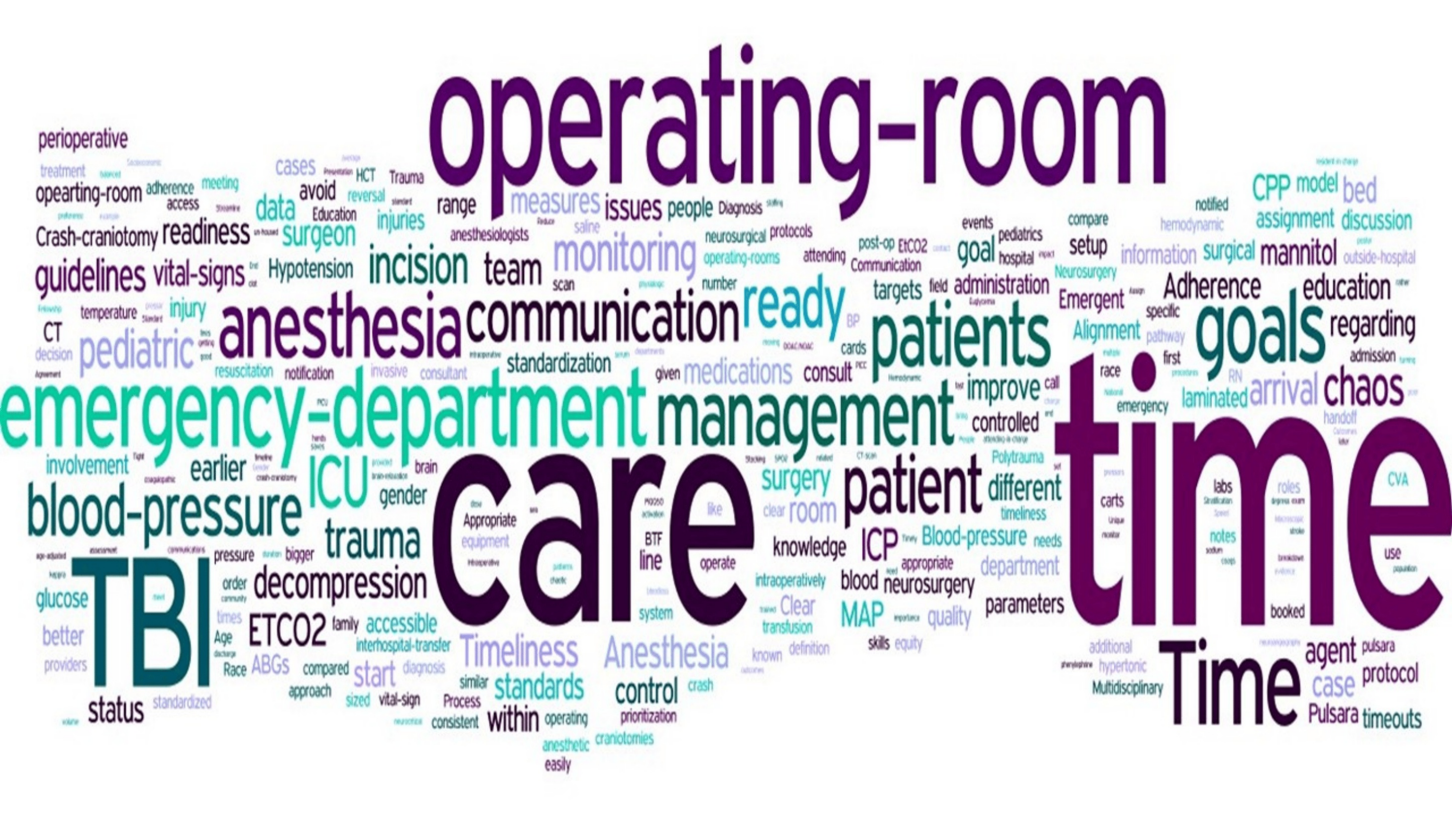
Perspectives in a word cloud Combined textual data of anesthesiology provider (attendings, residents, and nurse anesthetists) perspectives in a word cloud representation utilizing "Wordle," a free, online, artificial intelligence software.

Twenty-three anesthesiology providers responded to the four-question survey (13% response rate) and 27 anesthesiology providers participated in the two focus groups (10-17 anesthesiology attending physicians, resident physicians, and nurse anesthetists per meeting [15% response rate]). Initially, textual analysis resulted in more than 2,000 occurrences of 756 unique words and 9 unique numbers. Based on OEC’s 99 most common English words, 26.72% (n = 550) words were eliminated, leaving a total of 1,551 words. Although the word “time” is identified as common by the OEC, the high volume of repeats of this word, its similarity to “timeliness” or “timely,” and the importance of “timely” care per AHRQ domains of healthcare quality, the word "time" was retained within the analysis. Of the 1,551 total words, 450 words were unique and used once, whereas 50 unique words were repeated 54 times in survey and group discussions of TBI anesthesia care with anesthesiology providers (attending physicians, resident physicians, and nurse anesthetists) (Figure 2). Combining textual data of anesthesiology provider perspectives in a word cloud representation utilizing “Wordle” (free, online, artificial intelligence software), the word cloud of frequency of repeated words, showed “time” as the graphically largest word (Figure 3).

**Figure 4:**
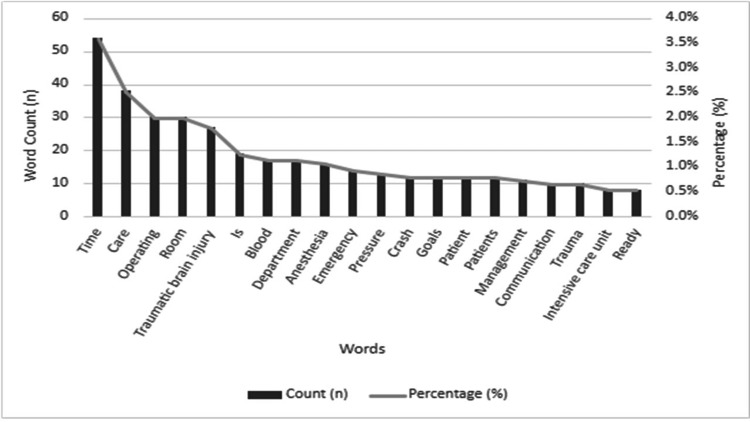
Top 20 words after common word exclusion The leading 20 words by count (n) and percentage (%) from all textual data of anesthesiology provider (attendings, residents, and nurse anesthetists) perspectives after exclusion of common words based on Oxford English Corpus's 99 most common English words.

The 20 most frequent unique words were identified in the textual data after exclusion of the most common English words (Figure 4). The leading 20 words by count [n] and percentage [%] from all textual data of anesthesiology provider perspectives after exclusion of common words based on Oxford English Corpus’s 99 most common English words on word count analysis were “time” (n = 54, 3.8%), “care” (n = 38, 2.5%), “operating” (n=30, 2%), “room” (n=30, 2%), “TBI” (n=28, 1.8%), “is” (n=19, 1.3%), “blood” (n=18, 1.2%), “department” (n=18, 1.2%), “anesthesia” (n=17%, 1.1%), “emergency” (n=14, 0.9%), “pressure” (n=13, 0.8%), “crash” (n=11, 0.7%), “goals” (n=11, 0.7%), "patient” (n=11, 0.7%), “patients” (n =11, 0.7%), “management” (n=10, 0.6%), “communication” (n=10, 0.6%), “trauma” (n=10, 0.6%), “intensive care unit” (n=9, 0.4%), and “ready” (n=9, 0.4%).

**Figure 5:**
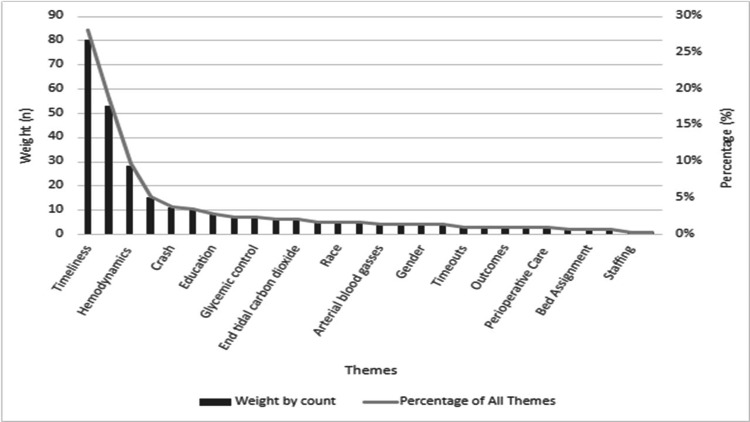
Content categories by weight and percentage Identified content categories from inductive content analysis of anesthesiology provider (attendings, residents, and nurse anesthetists) perspectives from survey and focus groups by weighted count and percentage.

**Figure 6:**
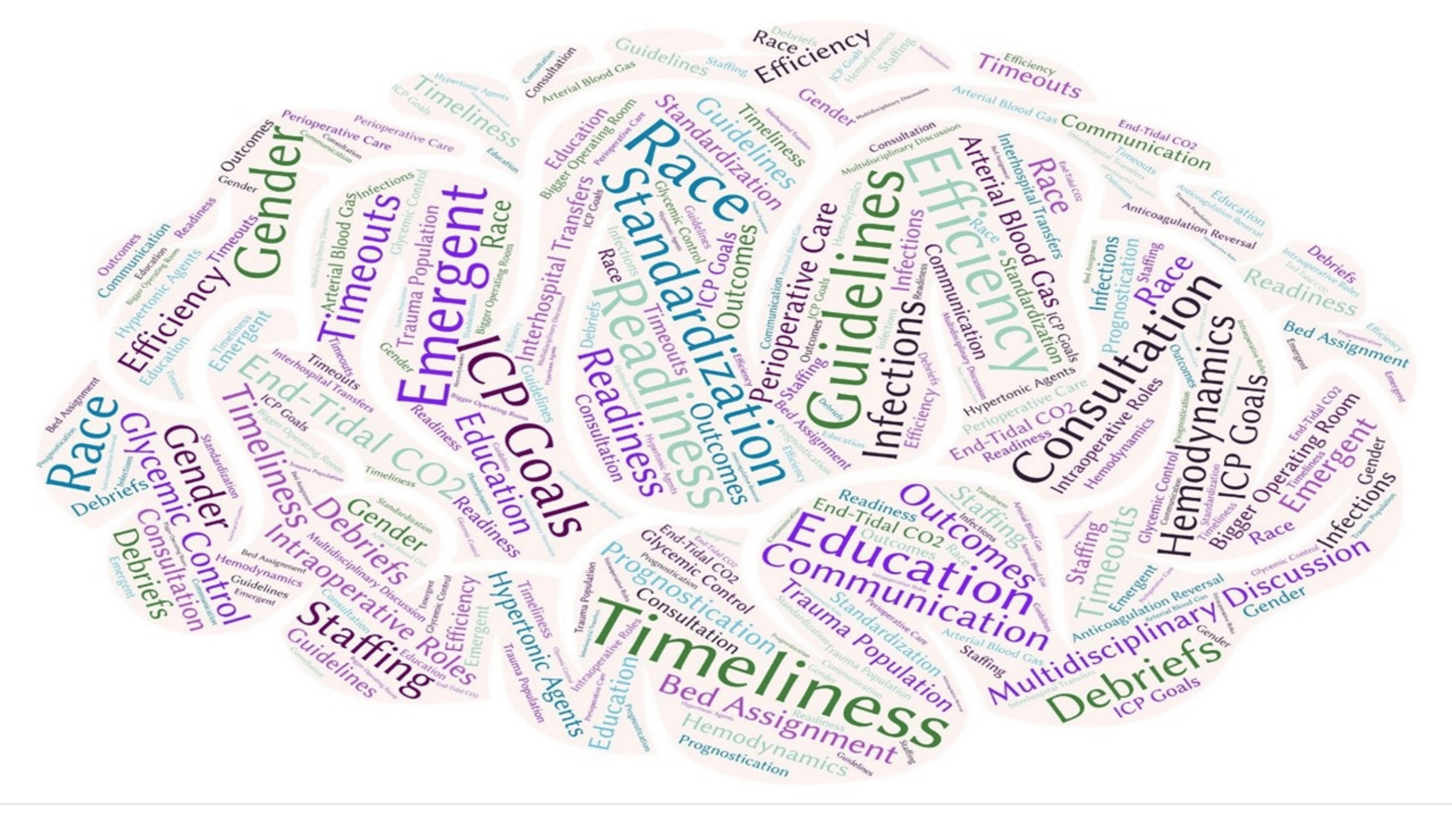
Word cloud representation of identified themes Figure of identified themes based on inductive content analysis of anesthesiology provider (attendings, residents, and nurse anesthetists) perspectives in a word cloud representation.

Further inductive analysis produced 28 different content categories with “timeliness” (28.1%), followed by “standardization” (18.6%), “hemodynamics” (8.5%), “communication” (5.0%), “crash” (3.2%), “readiness” (2.5%), “education” (2.4%), “consults” (2.4%), “glycemia control” (2.4%), “hypertonic agents” (2.3%), “end-tidal carbon dioxide” (2.3%), “intraoperative roles” (2.2%), “race” (2.2%), “intracranial pressure” (2.1%), “arterial blood gas” (2.0%), “trauma population” (2.0%), “gender” (2.0%), “multidisciplinary discussion” (2.0%), “time outs” (1.9%), “larger operating room” (1.9%), “outcomes” (1.8%), “debriefs” (1.7%), “perioperative care” (1.5%), “interhospital transfer” (1.0%), “bed assignment” (0.8%), “infections” (0.6%), “staffing” (0.2%), and “anticoagulation reversal” (0.2%) as the referenced themes within textual data from inductive content analysis of anesthesiology provider perspectives from survey and focus groups by weighted count [n] and percentage [%] (Figure 5). These themes were then re-visualized in another word cloud based on inductive content analysis of anesthesiology provider perspectives in a word cloud representation, which showed that the largest word groups were as follows: 1) “timeliness,” “emergent,” “guidelines,” “efficiency,” “race,” “ICP goals,” “readiness,” 2) “gender,” “standardization,” “education,” “consultation,” “debriefs,” and 3) “hemodynamics,” “timeout,” “staffing,” “infection,” “end-tidal CO2” (Figure 6).

**Figure 7:**
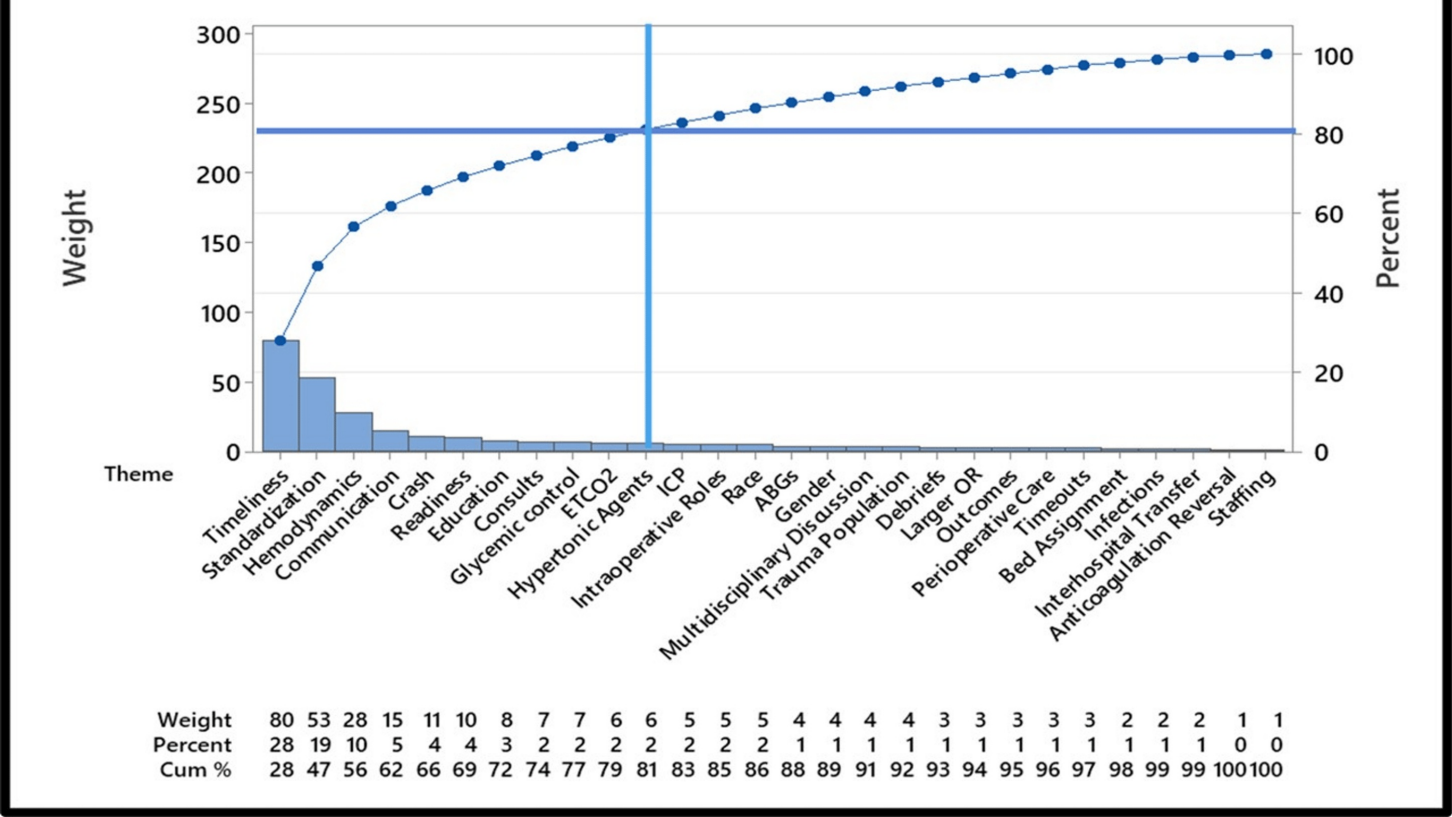
Pareto chart of themes Pareto chart of themes identified by inductive content analysis of all textual data obtained from anesthesiology provider (attendings, residents, nurse anesthetists) perspectives survey and focus group discussion (after exclusion of Oxford English Corpus's 99 most common English words. ETCO2, end-tidal carbon dioxide; ICP, intracranial pressure

Additional thematic analysis yielded classification of themes into 11 structure-type, 15 process-type, two outcome-type, and no balance-type QI categories. Table 1 shows the identified TBI anesthesia care themes classified by quality measure categories. The Pareto diagram of themes indicated “timeliness,” “standardization,” “hemodynamics,” “communication,” “crash,” “readiness,” “education,” “glycemic control,” “end-tidal CO2,” and “hypertonic agents” as important based on the 80-20 rule (Figure 7).

**Table 1:** Themes by quality measure categories TBI, traumatic brain injury; ETCO2, end-tidal carbon dioxide; ABG, arterial blood gas; ICP, intracranial pressure

Structure	Process	Outcome	Balance
Standardization of care	Timeliness	Outcomes	
Communication	Hemodynamic management	Infections	
“Crash” diagnosis	Glycemic control		
Readiness	ETCO_2_		
TBI education	ABG		
Neurosurgery consultation	ICP monitoring/management		
Neuro-prognostication/Multidisciplinary discussion	Hypertonic agents		
Larger operating room size	Defined intraoperative roles		
Interhospital transfers	Race (equity)		
Staffing	Gender (equity)		
Perioperative care	Trauma population /other injuries		
	Timeouts		
	Debriefs		
	Anticoagulation reversal		
	Bed assignment		

**Figure 8:**
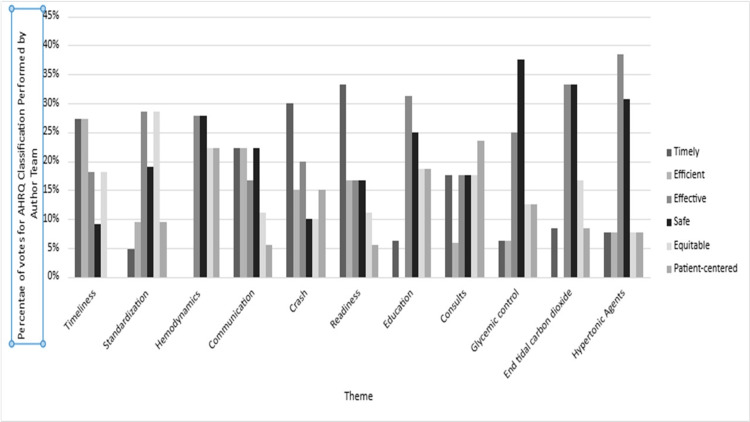
AHRQ domain rankings by theme Seven-member clinician author group rankings of top 20% of traumatic brain injury anesthesia care themes by AHRQ domains. AHRQ, Agency for Healthcare Research and Quality

**Figure 9:**
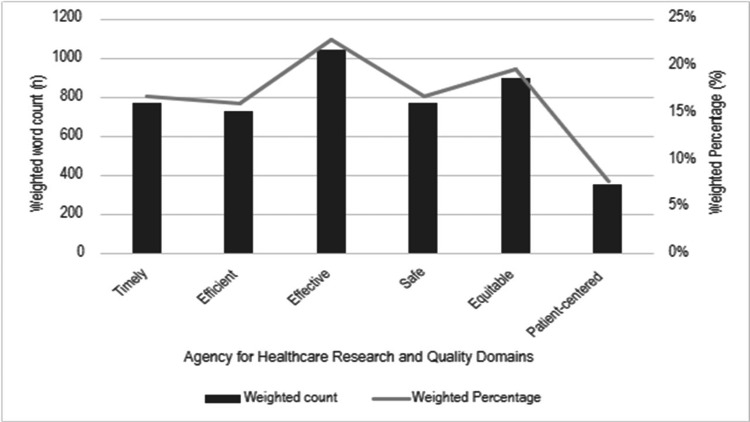
Theme classification into AHRQ domains by weighted count and percentage Weighted percentage of the top 20% of identified themes by the six AHRQ domains. The most identified themes were within the "effective, equitable, timely, and safe" domains. AHRQ, Agency for Healthcare Research and Quality

Figure 8 shows the seven-member clinician author team rankings of the leading 20% of identified themes according to AHRQ domains. Figure 9 shows the breakdown of weighted percentage of the top 20% of identified themes by the six AHRQ domains, with most identified themes being within the “effective, equitable, timely, and safe” domains.

## Discussion

Anesthesiology practice lacks a strong evidence base to guide emergency anesthesia care for patients with TBI. Additionally, there are no standard QI metrics in TBI anesthesia care. We conducted this study to better understand provider perspectives regarding priorities in emergent TBI anesthesia care. We learned that word cloud analysis was an efficient and visually detailed way to examine provider perspectives on TBI anesthesia care through main topics or themes within textual data [17,18]. Results provide new information to guide intervention development to optimize anesthesia care for patients with TBI.

Since deductive reasoning is limited by a lack of prior research, utilizing mixed methods including inductive content analysis and Pareto diagram helped identify high-priority content categories suitable for the future development of quality indicators and improvement metrics. Use of mixed methods evaluation and triangulation by applying word cloud and inductive reasoning identified many specific opportunities for improving emergent TBI anesthesia care. We have identified and classified some common themes on how to improve TBI anesthesia care, which showed variability in AHRQ domain categorization that merits further examination. The use of inductive reasoning in an area of care where evidence is lacking to support deductive reasoning and inclusion of provider input from physicians, trainees, and nurse anesthetists are strengths of this work. We utilized a focus group approach, which resulted in identification of priorities, and subjective analysis resulted in content category determination from the textual data.

Quantitative analysis of subjective anesthesiology provider input converted into word clouds that informed subsequent qualitative inductive reasoning was effective for identifying common areas for improving care of patients with TBI as determined by anesthesia providers. Our use of mixed methods analysis of provider input in areas of care where evidence limits deductive reasoning may be a desirable option for the identification of quality indicators and/or future investigation of ways to improve TBI anesthesia care and outcomes. This work is preliminary, single-center, and small in sample size, potentially limiting the generalizability to other institutions that provide emergency anesthesia care for patients with TBI. The problem of sampling bias from our small sample size and therefore replicability could be mitigated by increasing and broadening our pool of providers to include other organizations. Our focused stem (emergent crash craniotomy) and survey questions (Appendix) may have limited responses by our sample of providers resulting in few and non-specific outcome-type measures and no balance-type quality measures. Despite these limitations, this work presents important background for intervention development, which is underway to optimize emergent anesthesia care in these patients.

Existing national guidelines for the care of adults with severe TBI provide evidence-based recommendations, largely only relevant to the intensive care unit. Hence, there is an urgent need to develop an evidence base for emergency anesthesia care for patients with TBI, whether they receive neurosurgical care or anesthesia care for polytrauma. While there is overlap in areas of clinical practice, such as blood pressure management and/or management of intracranial pressure, the compendium is devoid of recommendations for anesthesia care, potentially resulting in large variations in anesthesia care for patients with TBI. The lack of anesthesia research coupled with the lack of standard anesthesia care benchmarks leaves anesthesia providers without best practice guidance. This study, while formative, provides input regarding priorities of anesthesiology practice during emergency care of patients with TBI.

## Conclusions

Determining local priorities for emergent TBI anesthesia care was feasible and resulted in themes for inclusion in intervention development. We identified important structure, process, and outcome that may be used as measures for future intervention evaluation. Word cloud, inductive reasoning, and the use of the Pareto diagram identified many opportunities for improving emergent TBI anesthesia care. Lessons learned from this work may help develop strategies to improve TBI anesthesia care through development of an anesthesia-specific TBI clinical care pathway to improve patient outcomes and develop focused areas for future research. This novel mixed methods approach can be utilized to better understand provider priorities pertaining to variably resourced trauma centers and to examine if our local findings are generalizable regarding anesthesiology provider identified gaps in TBI anesthesia care. Development of a clinical care pathway that aligns stakeholder buy-in and addresses priorities should decrease variations in emergent TBI anesthesia care.

## Appendices

Anesthesia Care of Patients With Traumatic Brain Injury for Emergent Craniotomy

Focused stem and survey questions

Anesthesia Care of Patients with Traumatic Brain Injury For Emergent Craniotomy: A 40-year-old patient with subdural hematoma after a motor vehicle crash is coming to the OR for a crash craniotomy. The first three questions refer to this case.

Have you taken care of a patient like this before?                                                                               __________________________________________

You are taking care of a patient similar to the case described above. What are one or more high-priority quality improvements you would recommend for our perioperative TBI care?                                                     __________________________________________

You are taking care of a patient similar to the scenario above. What are some quality measures we could use to evaluate our perioperative TBI care?          __________________________________________

We are trying to develop equity measure for perioperative TBI care. If you are taking care of a patient similar to the scenario above, what equity measures should we use to evaluate our perioperative TBI care?                                                                             ________________________________________

This question refers to pediatric TBI.

Are there opportunities to improve perioperative TBI care of infants and children, beyond what you have answered above?                                                                                 _________________________________________

Do you want to help with the work on rolling out these improvements? Yes, No, Maybe

Please provide us with your name and e-mail address __________________________________________

Please identify your role:

___Anesthesiology Faculty

___Anesthesiology Resident

___Certified Registered Nurse Anesthetist

___Certified Anesthesia Technician

## References

[1] Alinani A, Mills B, Gause E, Vavilala MS, Lele AV (2022). National Institutes of Health Clinical Research Funding and all-cause in-hospital traumatic brain injury-related mortality. Cureus.

[2] Berwick D, Bowman K, Matney C (2022). Traumatic Brain Injury: A Roadmap for Accelerating Progress.

[3] Jeremitsky E, Omert L, Dunham CM, Protetch J, Rodriguez A (2003). Harbingers of poor outcome the day after severe brain injury: hypothermia, hypoxia, and hypoperfusion. J Trauma.

[4] Sharma D, Brown MJ, Curry P, Noda S, Chesnut RM, Vavilala MS (2012). Prevalence and risk factors for intraoperative hypotension during craniotomy for traumatic brain injury. J Neurosurg Anesthesiol.

[5] Carney N, Totten AM, O'Reilly C (2017). Guidelines for the Management of Severe Traumatic Brain Injury, Fourth Edition. Neurosurgery.

[6] Algarra NN, Lele AV, Prathep S, Souter MJ, Vavilala MS, Qiu Q, Sharma D (2017). Intraoperative secondary insults during orthopedic surgery in traumatic brain injury. J Neurosurg Anesthesiol.

[7] UW Medicine - Harborview Medical Center: UW Medicine Level 1 (2023). UW Medicine - Harborview Medical Center: UW Medicine Level 1 Trauma services. https://www.uwmedicine.org/sites/stevie/files/2018-11/Provider-Resources-Trauma-HMC-Trauma-Services-Fact-Sheet-new2.pdf.

[8] Harris PA, Taylor R, Minor BL (2019). The REDCap consortium: Building an international community of software platform partners. J Biomed Inform.

[9] Harris PA, Taylor R, Thielke R, Payne J, Gonzalez N, Conde JG (2009). Research electronic data capture (REDCap)--a metadata-driven methodology and workflow process for providing translational research informatics support. J Biomed Inform.

[10] (2023). Word counter: Word Count Tool. https://www.wordcounttool.com/.

[11] (2023). The OEC: Facts about the Language. https://web.archive.org/web/20111226085859/http://oxforddictionaries.com/words/the-oec-facts-about-the-language.

[12] (2023). Wrdl: Wordle. https://www.wordle.net/.

[13] Vears DF, Gillam L (2022). Inductive content analysis: a guide for beginning qualitative researchers. Focus Health Prof Educ.

[14] (2024). Word Art: Word Cloud Generator. https://wordart.com/.

[15] Silver SA, Harel Z, McQuillan R (2016). How to begin a quality improvement project. Clin J Am Soc Nephrol.

[16] (2024). Six Domains of Healthcare Quality. https://www.ahrq.gov/talkingquality/measures/six-domains.html.

[17] McNaught C, Lam P (2010). Using Wordle as a supplementary research tool. Qualitative Rep.

[18] Douma L, Steverink N, Hutter I, Meijering L (2017). Exploring subjective well-being in older age by using participant-generated word clouds. Gerontologist.

